# Vitamin D deficiency in a high secure forensic psychiatry hospital: A clinical audit and service evaluation

**DOI:** 10.1192/bjo.2021.123

**Published:** 2021-06-18

**Authors:** George Gillett, Samantha Chudleigh-Warren, Benedict Hardy, Charles Robert Gordon

**Affiliations:** 1 IoPNN King's College London; 2 Specialty Doctor in Psychiatry, Broadmoor Hospital; 3 Consultant Forensic Psychiatrist, Broadmoor Hospital; 4 Specialty Doctor in Psychiatry, Broadmoor Hospital

## Abstract

**Aims:**

To assess concordance with guidelines on monitoring vitamin D levels and prescribing prophylaxis or replacement. To assess the association between the implementation of local guidelines and prevalence of vitamin D deficiency.

**Background:**

Vitamin D deficiency is associated with various adverse health outcomes including osteoporosis, fractures and myalgia. Most recently, vitamin D deficiency has been hypothesised as a risk factor for severe COVID-19 infection. Risk factors for vitamin D deficiency include incarceration, ethnicity, diet and a diagnosis of psychiatric disorder. Vitamin D deficiency is known to be prevalent among individuals within forensic mental health institutions.

Local Trust guidelines advise that vitamin D levels should be checked within one-month of hospital admission, followed by checks at three-monthly intervals. Recommendations for prescribing depend on patients’ vitamin D levels; deficient (<25nmol/L), insufficient (25 < 50nmol/L) or adequate (50-150nmol/L). We assessed concordance with these guidelines at Broadmoor Hospital, UK.

**Method:**

Medical records, laboratory results and drug charts were assessed for a total of 75 patients across 15 wards. Data were collected using a standardised audit tool, including; date of admission, admission vitamin D level, most recent vitamin D level and the dose and frequency of vitamin D prescribed.

**Result:**

76.4% of patients had their vitamin D levels checked within one month of admission. 66.7% of patients had their vitamin D checked within the last 3 months. For patients with an admission vitamin D level recorded, 43.6% had deficient vitamin D levels, 43.6% had insufficient levels and 12.7% had adequate levels. For patients with a more recent serum vitamin D level, 14.5% had deficient levels, 38.7% had insufficient levels and 46.8% had adequate levels. For patients with a documented serum vitamin D level, 21.4% were prescribed the correct dose, 22.9% were under-dosed, 14.3% were over-dosed and 41.4% received no dose where guidelines suggested they should.

**Conclusion:**

Comparison of admission and most recent vitamin D levels suggests a general improvement in prevalence of vitamin D deficiency associated with the implementation of local guidelines. However, we identify significant areas for improvement. A substantial proportion of patients lacked admission or regular monitoring of vitamin D levels and a substantial proportion of patients were under-dosed or received no dose where guidelines suggested they should have. We propose that better concordance with guidelines may improve clinical outcomes further. This may prove especially important during the COVID-19 pandemic, given a potential association between vitamin D deficiency and severity of respiratory infection.

